# Osteoarthritis or arthritis? Toward understanding of primary Sjögren’*s* syndrome patients with arthralgia

**DOI:** 10.1186/s13018-023-03513-1

**Published:** 2023-01-16

**Authors:** Ronglin Gao, Jincheng Pu, Zhenzhen Wu, Jianping Tang, Xuan Wang

**Affiliations:** Department of Rheumatology and Immunology, Tongji Hospital, School of Medicine, Tongji University, No. 389 Xincun Road, Shanghai, 200065 China

**Keywords:** Primary Sjögren’*s* syndrome, Osteoarthritis, Arthritis, Joint pain, Metabolism

## Abstract

**Objective:**

To identify primary Sjögren’*s* syndrome (pSS) patients with arthralgia at risk for osteoarthritis (OA) or arthritis.

**Methods:**

This study included 368 pSS patients admitted to a mono-centric from March 2010 to December 2020. Patients were divided into groups according to whether complicated with OA or arthritis. Data were analyzed to determine the differences in demographical characteristics, symptoms, and laboratory examination.

**Results:**

The involvement of the OA joints was predominately knee and spine sites (including cervical and lumbar spine degeneration). When diagnosing arthritis, it was mainly peripheral symmetric polyarthritis, the most affected sites were the interphalangeal and metacarpophalangeal joints. There were significant differences in age, disease duration, uric acid (UA), and total cholesterol (TC) between pSS-OA and pSS-nOA patients (*P* < 0.050). Logistic regression analysis showed that age (OR = 1.965; *P* = 0.009) and joint pain (OR = 3.382; *P* < 0.001) were dangerous factors associated with OA. Interestingly, although the level of UA, TC, and triglycerides (TG) was shown to be positive with OA, there was no statistical significance after the OR was computed in the four-cell table. In pSS-arthritis, EULAR Sjögren'*s* syndrome disease activity index (ESSDAI) (*P* = 0.011), the frequency of joint pain (*P* < 0.001), and muscular involvement (*P* = 0.037) were higher than non-arthritis group. In pSS patients only presenting with joint pain, arthritis patients had higher ESSDAI and system involvements, but lower UA and TG levels compared with OA group (*P* < 0.050).

**Conclusion:**

In pSS patients with arthralgia, OA accounted for the majority. pSS patients with advanced age and more pronounced metabolic characteristics, such as elevated blood lipids and uric acid, was a key factor in groups at risk for OA. However, arthritis patients had higher rates of dry mouth and eye, higher disease activity, antibodies positive, and more organs damage. In the future, it may be necessary to be more cautious in the diagnosis of joint manifestations in pSS patients in order to make the appropriate treatments.

**Supplementary Information:**

The online version contains supplementary material available at 10.1186/s13018-023-03513-1.

## Introduction

Primary Sjögren’*s* syndrome (pSS) is a systemic autoimmune disease with different incidences in different countries and regions, with an average prevalence of 0.5–1%. Women are more likely to develop pSS than men, especially postmenopausal women, at a rate of about 9:1. T lymphocyte- and B lymphocyte-mediated immune abnormalities, infection, genetics, inflammation, sex hormones, and other factors were involved in the occurrence and development of diseases [1, 2]. It not only targets exocrine glands with dryness of the mouth and eyes due to immune cell infiltration and also involves a variety of extraglandular manifestations, such as lung, joint and blood system, creating a chronic inflammatory environment for the body and causing progressive damage to organ functions [3]. We know pSS patients are at an increased risk of lymphoma clinically due to active B-cell mechanisms. Approximately 5% of pSS patients develop lymphoma, mucosa-associated lymphoid tissue lymphomas (MALTLs) constituted the majority of lymphomas. What’*s* worse, the rate can be as high as 16.8% during long follow-up at a referral center and lymphoma may invade one or more external nodes throughout the body, resulting in excessive mortality from pSS [4]. In addition, articular manifestations, including joint pain with or without arthritis, are the most common extraglandular manifestations [5, 6]. HarmonicSS (https://cordis.europa.eu/project/id/731944), a large international cohort of patients looking at the clinical presentation of pSS, found that 64% of patients had arthralgia and 16.6% had arthritis. Involvement in the peripheral joints is symmetrical in about half of cases, with the metacarpophalangeal (MCP), proximal interphalangeal (PIP), and wrist joints being commonly involved joints, but knees and ankles may also be affected [7, 8]. Another study showed that approximately 30–60% of pSS patients were suffering from articular manifestations (AMs) which was associated with multiple system involvement [9], as well as several studies reporting long-term follow-up of pSS patients with rare rheumatoid arthritis (RA) development [9–11]. However, there is no further in-depth research on the identification of pSS-related arthralgia symptoms, whether there are other possibilities except for arthritis.

We know that the other major cause of joint pain is osteoarthritis (OA), the most common arthritis in elderly individuals, which is associated with the development of several autoimmune diseases as degenerative changes, such as RA and connective tissue disease (CTD). A study showed that the prevalence of OA and the number of years lost by disability had all increased significantly in terms of time [12]. OA brings serious harm to physiology and psychology, causing an increase in the incidence of joint pain and deformities, mobility disorders, and depression in patients [13, 14]. In the past, OA was a degenerative disease, which was usually described as a non-inflammatory disease. Now, several risk factors, such as female, obesity, metabolic syndrome, heredity, and age, are independently associated with OA [15, 16]. A cohort study consisting of 63,626 individuals reported a 2.75-fold higher risk of OA in RA patients compared to non-RA patients, suggesting the risk of OA in other autoimmune diseases [17]. In a study including 124 patients with systemic sclerosis (SSc), 19% had the typical presentation of erosive hand OA, which may relate to the course of the disease, organ involvement, and SSc-related antibodies, reflecting the underlying pathogenesis. Erosive OA is more common in patients with SSc than in the general population [18]. Also, a joint performance study indicated 7 of 38 progressive systemic sclerosis (PSS) had erosive distal interphalangeal joints OA [19]. It can be seen that there is a strong correlation between OA and CTD, but the connection needs to be further clarified.

Recently, we have seen a significant increase in the number of patients with pSS combined with OA in daily work. A controlled study pointed out that patients with pSS had an increased incidence of hand OA as compared to patients with systemic lupus erythematosus (SLE) and was found that the frequency of erosive hand OA in pSS patients was significantly higher than that of the general population [20]. Nowadays, the detailed clinical characteristics between pSS with OA and arthritis, whose most common symptom is joint pain, are not studied well. Therefore, we discuss arthralgia in pSS patients, hoping to clarify the characteristics of this population, to facilitate differential diagnosis, and to provide some clues for the identification of different clinical phenotypes about arthralgia.

## Material and method

### Patients and study design

A total of 368 patients with pSS registered in the Department of Rheumatology and Immunology of Shanghai Tongji Hospital were retrospectively analyzed between March 2010 and December 2020. Patients were considered eligible for the study if they fulfilled the diagnostic criteria of pSS proposed by the American College of Rheumatology /European League Against Rheumatism (ACR/EULAR) in 2002/2016 [21, 22]. There was no clinical or immunological evidence of other connective tissue diseases and patients with the diagnosis of inflammatory arthritis (gout) were excluded. Diagnostic tests for SS, labial gland biopsy, salivary gland emission computed tomography (ECT), lacrimal gland examination, were applied according to the recommendations of the ACR/EULAR Group [22]. Arthralgia mainly was based on patients' self-reported symptoms and defined when the visual analog score (VAS) that is the score of 0–10 numerical scales assessing for joint pain was greater than or equal to 1 point. Arthritis referred to the inflammation of one or more joints, including the joints of the hands, feet, wrists, ankles, etc., which was manifested by joint pain, swelling, redness, morning stiffness for more than 30 min, or elevated erythrocyte sedimentation rate (ESR), imaging findings suggesting synovitis, but excluding RA, ankylosing spondylitis, gout, and other inflammatory arthritis diseases. To verify the complications of OA which referred to the diagnostic criteria of Chinese guidelines for diagnosis and treatment of osteoarthritis (2018 Edition), X-ray, computed tomography (CT), and magnetic resonance imaging (MRI) findings of the joints’ degenerative changes, and the sign and symptoms of joint were analyzed by two experienced rheumatologists [23]. Patients were grouped according to whether they had OA or arthritis.

### Clinical variables and laboratory indicators

All the patients included in this study were assessed for demographical characteristics, symptoms, and laboratory examinations. According to the EULAR SS Patient Reported Index (ESSPRI) and the EULAR SS Disease Activity Index (ESSDAI), two methods for assessing disease activity indices in pSS, the validity of which has been well corroborated, defining the patient'*s* symptom and comorbidity status [24, 25]. Items included the following aspects: gender, age, disease activity, disease duration, and comorbidities (pulmonary disease, gastrointestinal diseases, renal manifestations, nervous system and blood system, etc.). Symptoms involved were dry eyes, dry mouth, parotid gland swelling, joint pain, muscle involvement, Raynaud'*s* phenomenon, etc. Disease activity was evaluated by following the ESSDAI [24]. Meanwhile, laboratory examination consisted of various aspects, including routine blood test, liver and kidney function, heart function, thyroid function, blood glucose, lipid metabolism, etc. Immunologic tests were performed to determine the autoimmune-related antibodies, rheumatoid factor (RF), anti-cyclic citrullinated peptide (CCP) antibody, complement factors (C3 and C4), immunoglobulin, etc. All laboratory tests were performed in the clinical chemistry and immunology laboratories of Shanghai Tongji Hospital.

### Data analysis

The information is stored in the medical record system of Shanghai Tongji Hospital. After two people entered and organized the patient-related data, a third person again verified the accuracy and completeness of the data to ensure that the clinical and laboratory test data were complete. Data were assessed to perform this statistical analysis using the statistical software packages IBM SPSS Statistics 26.0, R language, and GraphPad Prism 8.0. Categorical variables were expressed as numbers and percentages, and quantitative variables were expressed as mean ± standard deviation (SD). The traditional chi-square test, Mann–Whitney U test, and independent-sample t test were used to analyze the quality differences between different groups. Binary regression analysis was used to explore the relationship between two variables and calculate the odds ratio (OR) to assess the risk of each variable. Age, sex, and course of disease were matched between groups using R language, and the adjusted P value was calculated using Benjamini–Hochberg method. *p* < 0.05 indicated statistical significance, and the confidence interval (CI) was 95%.

## Result

Of the 368 patients enrolled, 162 had symptoms of arthralgia. Hundred and eighty-six patients had osteoarthritis and 41 had arthritis, a prevalence of 11.14%. Nearly half of the patients with OA had multiple joint involvements, predominately knee and spine (including cervical and lumbar spine degeneration) which were large, weight-bearing joints, followed by hip joints, hand joints (including interphalangeal and metacarpophalangeal joints), etc. When diagnosing arthritis, it was mainly peripheral symmetric polyarthritis, the most affected sites were the PIP joint and MCP joint (Table 1).

**Table 1:** Affected joints of osteoarthritis and arthritis

Joint (*n*, %)	Osteoarthritis	pSS-arthritis
	*n* = 186	*n* = 41
Hand	20(10.8)	
MCP		17(41.5)
DIP		11(26.8)
PIP		25(61.0)
Wrists	4(2.2)	10(24.4)
Elbows	7(3.8)	8(19.5)
Knee	86(46.2)	8(19.5)
Hips	29(15.6)	0(0.0)
Shoulders	20(10.8)	11(26.8)
Spine	111(59.7)	5(12.2)
Other	17(9.1)	10(24.4)

*MCP*: metacarpophalangeal; *DIP*: distal interphalangeal; *PIP*: proximal interphalangeal

### Characteristics of pSS patients with or without OA

Table 2 summarizes the demographic, clinical, and serological features of patients in the pSS-OA group (pSS patients with OA) and pSS-nOA group (pSS patients without OA). Our results found that there was 172 female (92.5%) in the pSS-OA group, which had a similar ratio as another group. Age and duration of disease in the pSS-OA were all longer than in the pSS-nOA group, and they are statistically significant (*p* < 0.050). In the pSS-OA group, the median score of ESSDAI was 4, lower than that of the pSS-nOA group, but there’*s* no significant difference between the two groups in most ESSDAI domains. The serological analysis showed that uric acid (UA) (*p* = 0.030) and total cholesterol (TC) (*p* = 0.025) in OA group were higher than in the nOA group. By contrast, we found that immune indexes, such as ANA positive, C4, IgG, IgA, and globulin levels, all were lower in pSS-OA group (*p* < 0.050). Neither anti-CCP antibodies nor RF was detectable differently in the serum samples (*p* > 0.050). We used binary regression analysis to analyze the relevant predictors of pSS combined with OA. Table2 also displays that pSS patients with seniority (OR = 1.965; *p* = 0.009) and joint pain (OR = 3.382; *p* < 0.001) were more likely to have OA. But the P values adjusted by the Benjamini-Hochberg method were mostly no longer statistically significant (Additional file 1: Table S1). After age, sex, and disease duration were matched, the variables for which there was a statistical difference before were no longer significant (Additional file 1: Table S2).

**Table 2:** Characteristics of pSS patients with or without OA

Variable	pSS-OA	pSS-nOA	*p* value^1^	OR	*p* value^2^
	*n* = 186	*n* = 182			
Female (*n*, %)	172(92.5)	166(91.2)	0.659		
Age (≥ 65 year) (*n*, %)	71(38.2)	41(22.5)	0.001	1.965	0.009
Disease duration (year) M (IQR)	5(2–10)	3(1–10)	0.034	1.023	0.112
Clinical manifestation (*n*, %)					
Dry mouth	164(88.2)	150(82.4)	0.119		
Dry eye	134(72.8)	121(66.9)	0.215		
Mouth ulcers	52(28.0)	37(20.3)	0.088		
Dental caries and dentures	114(61.3)	102(56.0)	0.308		
Swollen parotid gland	20(10.8)	20(11.0)	0.942		
Rash	34(18.3)	45(24.7)	0.133		
Renault phenomenon	22(11.8)	32(17.6)	0.119		
Joint pain	108(58.1)	54(29.7)	< 0.001	3.382	< 0.001
Fatigue	35(18.8)	43(23.6)	0.260		
ESSDAI score M (IQR)	4(2–8)	5(3–10)	0.006	0.965	0.098
ESSDAI domains (*n*, %)					
Constitutional	1(0.5)	1(0.5)	1.000		
Lymphadenopathy	8(4.3)	11(6.0)	0.451		
Glandular	22(11.8)	17(9.4)	0.461		
Articular	24(12.9)	17(9.4)	0.279		
Cutaneous	5(2.7)	13(7.1)	0.048	0.359	0.057
Pulmonary	35(18.8)	42(23.5)	0.278		
Renal	7(3.8)	6(3.4)	0.833		
Muscular	4(2.2)	3(1.6)	1.000		
Peripheral nervous system	6(3.2)	11(6.0)	0.199		
Central nervous system	0(0.0)	1(0.5)	0.495		
Hematological	109(58.6)	123(67.6)	0.075		
Biological	103(55.7)	114(64.8)	0.078		
Laboratory data					
Hemoglobin (g/L), *x* ± *s*	119 ± 14.8	115 ± 18.8	0.030	1.005	0.515
A/G	1.3 ± 0.3	1.2 ± 0.3	0.012	0.847	0.796
Globulin (g/L), *x* ± *s*	31.8 ± 7.5	34.5 ± 11.0	0.008	0.958	0.128
Albumin (g/L), *x* ± *s*	38.2 ± 6.0	37.1 ± 5.1	0.058		
K^+^ (mmol/L), *x* ± *s*	3.8 ± 0.3	3.7 ± 0.4	0.015	1.404	0.263
Uric acid (umol/L), M (IQR)	276(234.7–328.3)	261(218.0–317.0)	0.030	1.002	0.104
TC (mmol/L), M (IQR)	4.6(4.0–5.3)	4.5(3.7–4.9)	0.025	1.015	0.670
TG (mmol/L), M (IQR)	1.3(0.9–1.7)	1.3(0.9–1.4)	0.287		
HDL (mmol/L), M (IQR)	1.2(1.0–1.4)	1.2(0.9–1.3)	0.064		
FBG (mmol/L), *x* ± *s*	5.0 ± 1.0	4.9 ± 1.0	0.537		
C3 (g/L), *x* ± *s*	1.0 ± 0.2	0.9 ± 0.2	0.256		
C4 (g/L), *x* ± *s*	0.2 ± 0.1	0.19 ± 0.1	0.004	4.213	0.430
ANA positive (*n*, %)	113(61.1)	131(72.0)	0.027	0.676	0.120
Anti-SSA positive (*n*, %)	107(57.8)	123(67.6)	0.054		
Anti-SSB positive (*n*, %)	48(25.9)	53(29.1)	0.497		
Anti-CCP positive (*n*, %)	5(3.1)	4(2.8)	0.868		
Increased RF (*n*, %)	38(22.9)	45(28.3)	0.265		
IgA (g/L), *x* ± *s*	2.9 ± 1.4	3.3 ± 2.9	0.049	0.894	0.060
IgM (g/L), *x* ± *s*	1.4 ± 1.4	1.6 ± 2.2	0.444		
IgG (g/L), *x* ± *s*	15.5 ± 6.0	16.7 ± 7.2	0.035	1.029	0.357
IgG4 (g/L), *x* ± *s*	0.6 ± 1.8	0.5 ± 0.8	0.696		
Increased CRP (*n*, %)	15(8.3)	23(13.3)	0.133		
Increased ESR (*n*, %)	51(28.3)	60(36.1)	0.121		
Decreased 25-(OH)D (*n*, %)	28(17.5)	18(12.5)	0.226		

*OA*: osteoarthritis; *A/G*: albumin/globulin; *UA*: uric acid; *TC*: total cholesterol; *TG*: triglycerides; *HDL*: high density lipoprotein; *FBG*: fasting blood glucose; *C3*: complement 3; *C4*: complement 4; *ANA*: antinuclear antibodies; *CCP*: cyclic citrullinated peptide; *RF*: rheumatoid factor; *CRP*: C-reactive protein; *ESR*: erythrocyte sedimentation rate; *25-(OH) D*: 25-hydroxy vitamin D

### Characteristics of pSS patients with arthritis

Table 3 shows pSS patients with arthritis had a median ESSDAI score of 7(4–9.5), significantly higher than patients without arthritis with a median ESSDAI score of 4(2–10) (*p* = 0.011), and more organs were involved in ESSDAI domains. There were 38 cases (92.7%) and 28 cases (68.3%) with subjective xerostomia and xerophthalmia, and the incidence of subjective and objective xerostomia and xerophthalmia was similar in the two groups. Swollen parotid glands and joint pain (*p* < 0.001) were more common in people with arthritis. There was no difference in C-reactive protein (CRP), ESR, and RF positivity between the two groups (*p* > 0.050). In addition, the glucose and lipid metabolism, complement, globulin, and antibody were not associated with arthritis (*P* > 0.050). Similar with the OA group, most P values adjusted by Benjamini-Hochberg method were no longer statistically significant (Additional file 1: Table S3). After age, sex, and disease duration were matched, only arthralgia, A/G, and fasting blood glucose were statistically different between the two groups (*P* < 0.050) (Additional file 1: Table S4).

**Table 3:** Characteristics of pSS patients with or without arthritis

Variable	pSS with arthritis	pSS without arthritis	*p* value
	*n* = 41	*n* = 327	
Female (*n*, %)	38(92.7)	300(91.7)	1.000
Age (≥ 65 year) (*n*, %)	13(31.7)	99(30.3)	0.851
Disease duration (year) M (IQR)	4(1–8)	4(2–10)	0.173
ESSDAI score M (IQR)	7(4–9.5)	4(2–10)	0.011
Clinical manifestation (*n*, %)			
Dry mouth	38(92.7)	276(84.4)	0.159
Objective	21(91.3)	201(91.8)	1.000
Dry eye	28(68.3)	227(70.1)	0.817
Objective	12(85.7)	70(82.4)	1.000
Swollen parotid gland	3(7.3)	37(11.3)	0.611
Renault phenomenon	4(9.8)	50(15.3)	0.346
Joint pain	36(87.8)	126(38.5)	< 0.001
Lymphadenopathy	1(2.4)	18(5.5)	0.644
Cutaneous	3(7.3)	15(4.6)	0.704
Pulmonary	9(22.0)	68(21.0)	0.887
Renal	2(4.9)	11(3.4)	0.972
Muscular	3(7.3)	4(1.2)	0.037
Peripheral nervous system	2(4.9)	15(4.6)	1.000
Central nervous system	0(0.0)	1(0.3)	1.000
Hematological system	25(61.0)	207(63.3)	0.772
Laboratory data			
Hemoglobin (g/L), *x* ± *s*	118 ± 17.3	117 ± 17.0	0.653
A/G	1.2 ± 0.3	1.2 ± 0.3	0.233
Globulin (g/L), *x* ± *s*	33.1 ± 6.3	33.2 ± 9.8	0.980
K^+^ (mmol/L), *x* ± *s*	3.8 ± 0.3	3.8 ± 0.4	0.059
Uric acid (umol/L), M (IQR)	273(209–331)	267(230–316)	0.881
TC (mmol/L), M (IQR)	4.4(3.9–5.3)	4.5(3.8–5.3)	0.951
TG (mmol/L), M (IQR)	1.1(0.8–1.7)	1.2(0.9–1.7)	0.246
HDL (mmol/L), M (IQR)	1.1(0.9–1.6)	1.2(0.9–1.4)	0.817
FBG (mmol/L), *x* ± *s*	4.8 ± 0.8	5.0 ± 1.0	0.297
C3 (g/L), *x* ± *s*	1.0 ± 0.2	0.9 ± 0.2	0.187
C4 (g/L), *x* ± *s*	0.2 ± 0.1	0.2 ± 0.1	0.435
ANA positive (*n*, %)	28(68.3)	216(66.3)	0.795
Anti-SSA (*n*, %)	24(58.5)	206(63.2)	0.563
Anti-SSB (*n*, %)	9(22.0)	92(28.2)	0.398
Anti-CCP (*n*, %)	1(2.9)	8(3.0)	1.000
Increased RF (*n*, %)	14(36.8)	69(24.0)	0.090
IgA (g/L), *x* ± *s*	2.9 ± 1.1	3.1 ± 2.4	0.540
IgM (g/L), *x* ± *s*	1.3 ± 0.9	1.5 ± 2.0	0.511
IgG (g/L), *x* ± *s*	16.8 ± 5.4	16.1 ± 6.8	0.573
IgG4 (g/L), *x* ± *s*	0.5 ± 0.6	0.6 ± 1.5	0.956
Increased CRP (*n*, %)	3(7.7)	35(11.1)	0.702
Increased ESR (*n*, %)	12(29.3)	99(32.5)	0.682
Decreased 25-(OH)D (*n*, %)	6(18.8)	40(14.7)	0.732

*A/G*: albumin/globulin; *UA*: uric acid; *TC*: total cholesterol; *TG*: triglycerides; *HDL*: high density lipoprotein; *FBG*: fasting blood glucose; *C3*: complement 3; *C4*: complement 4; *ANA*: antinuclear antibodies; *CCP*: cyclic citrullinated peptide; *RF*: rheumatoid factor; *CRP*: C-reactive protein; *ESR*: erythrocyte sedimentation rate; *25-(OH) D*: 25-hydroxy vitamin D

### Comparison between arthritis and OA

Excluding patients with coexisting OA and arthritis, among pSS patients presenting with joint swelling and pain, Table 4 shows the OA group was older than the arthritis group (*p* = 0.001), but the arthritis group had a higher median ESSDAI score (*p* = 0.005) and more significant involvement of other organ systems (*p* = 0.013). In addition, patients in the OA group tended to have higher levels of UA (*P* = 0.042) and TG (*p* = 0.040) compared with the arthritis group. No difference was found between RF and anti-CCP antibodies in the two groups (*p* > 0.050).

**Table 4:** Comparison of characteristics between arthritis and OA in pSS patients with arthralgia

Variable	pSS with arthritis	pSS with OA	*p* value
	*n* = 12	*n* = 84	
Female (*n*, %)	12(100.0)	78(92.9)	0.750
Age (year), *x* ± *s*	51 ± 14.5	62 ± 10.0	0.001
Disease duration (year) M (IQR)	5(1.8–7.8)	5(2–10)	0.341
ESSDAI score M (IQR)	7(4.8–8.5)	4(2.0–7.0)	0.005
Clinical manifestation (*n*, %)			
Dry mouth	12(100.0)	72(85.7)	0.351
Objective	7(100.0)	59(93.7)	1.000
Dry eye	9(75.0)	57(67.9)	0.868
Objective	5(100.0)	23(95.8)	1.000
Swollen parotid gland	1(8.3)	11(13.1)	1.000
Renault phenomenon	1(8.3)	10(11.9)	1.000
Lymphadenopathy	1(8.3)	3(3.6)	1.000
Cutaneous	3(25.0)	2(2.4)	0.013
Pulmonary	2(16.7)	15(17.9)	1.000
Renal	1(8.3)	2(2.4)	0.825
Muscular	1(8.3)	1(1.2)	0.107
Peripheral nervous system	1(8.3)	3(3.6)	1.000
Hematological	6(50.0)	53(63.1)	0.579
Laboratory data			
Hemoglobin (g/L), *x* ± *s*	123 ± 16.5	121 ± 12.4	0.590
A/G	1.2 ± 0.3	1.3 ± 0.3	0.428
Globulin (g/L), *x* ± *s*	32.6 ± 6.5	31.9 ± 6.1	0.696
UA (umol/L), M (IQR)	217(163.8–300.5)	284(230.0–335.0)	0.042
TC (mmol/L), M (IQR)	4.7(3.9–5.5)	4.8(4.0–5.4)	0.934
TG (mmol/L), M (IQR)	0.9(0.8–1.1)	1.3(0.9–1.7)	0.040
HDL (mmol/L), M (IQR)	1.1(1.0–1.6)	1.2(1.0–1.4)	0.980
FBG (mmol/L), *x* ± *s*	4.8 ± 0.5	5.0 ± 1.3	0.533
C3 (g/L), *x* ± *s*	1.0 ± 0.2	1.0 ± 0.2	0.741
C4 (g/L), *x* ± *s*	0.2 ± 0.1	0.2 ± 0.1	0.166
ANA positive (*n*, %)	10(83.3)	50(60.2)	0.219
Anti-SSA (*n*, %)	9(75.0)	50(60.2)	0.505
Anti-SSB (*n*, %)	3(25.0)	22(26.5)	1.000
Anti-CCP (*n*, %)	1(9.1)	3(4.2)	1.000
Increased RF (*n*, %)	4(33.3)	16(22.2)	0.638
IgA (g/L), *x* ± *s*	2.3 ± 0.9	3.0 ± 1.5	0.170
IgM (g/L), *x* ± *s*	1.1 ± 0.5	1.5 ± 1.4	0.418
IgG (g/L), *x* ± *s*	16.1 ± 5.4	15.0 ± 4.4	0.459
IgG4 (g/L), *x* ± *s*	0.5 ± 0.6	0.8 ± 3.0	0.791
Increased CRP (*n*, %)	1(8.3)	6(7.3)	1.000
Increased ESR (*n*, %)	2(16.7)	25(30.9)	0.503
Decreased 25-(OH)D (*n*, %)	3(37.5)	16(21.9)	0.584

*OA*: osteoarthritis; *A/G*: albumin/globulin; *UA*: uric acid; *TC*: total cholesterol; *TG*: triglycerides; *HDL*: high density lipoprotein; *FBG*: fasting blood glucose; *C3*: complement 3; *C4*: complement 4; *ANA*: antinuclear antibodies; *CCP*: cyclic citrullinated peptide; *RF*: rheumatoid factor; *CRP*: C-reactive protein; *ESR*: erythrocyte sedimentation rate; *25-(OH) D*: 25-hydroxy vitamin D

### Effects of metabolic factors on OA and arthritis

**Fig. 1:**
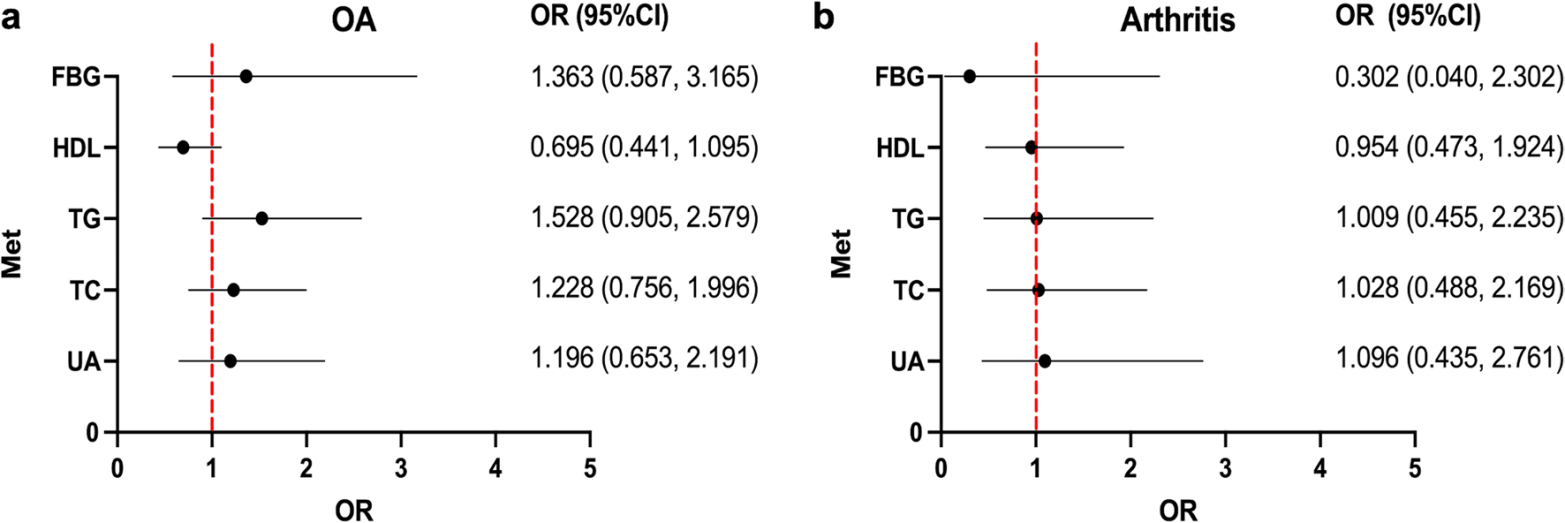
Association between Met components and joint changes in pSS patients. pSS-OA group versus pSS-nOA group (**a**); pSS-arthritis versus pSS-no arthritis group (**b**). pSS: primary Sjögren’*s* Syndrome; *OA*: osteoarthritis; Met: metabolism; *UA*: uric acid; *TC*: total cholesterol; *TG*: triglycerides; *HDL*: high density lipoprotein; *FBG*: fasting blood glucose

To investigate the possible role of metabolic factors on the occurrence of joint diseases, OR values were calculated, which are shown in Fig. 1. The risk of osteoarthritis in pSS patients was increased by increased blood lipids, uric acid, and fasting blood glucose, but all metabolic variables included in the model were not statistically significant. For arthritis, there was similarly no reliable evidence that metabolic factors contributed to disease development.

### Discussion

In this study, we found that more than half of pSS patients were suffering from joint pain and the most common cause was OA, especially knee OA, or joint involvement due to the disease itself. The older the pSS patient with joint pain and the longer the course of disease, the more likely it was to be combined with OA from the study. It is generally accepted that the main risk factor for osteoarthritis is age, and the prevalence and incidence of OA increase with age [14, 15]. Especially, between different types of OA, knee OA is more common in the aging population [26]. The function of important articular cartilage components, such as collagen type II, gradually diminishes, affecting articular cartilage stability with age. Aging can cause functional and structural changes in cells in the skeleton, leading to ongoing bone remodeling and bone destruction. Processes such as autophagy, inflammation, and oxidative stress may provide a critical link between chondrocyte senescence, survival, and OA [27, 28]. In addition, we found an association between OA and blood lipids. Compared with patients without OA, TG and TC levels were elevated and became risk factors for the development of the disease. A positive dose–response relationship between TC levels and the risk of KOA in clinical patients was demonstrated in a Chinese cohort [29]. In contrast, in other studies of the association between metabolic syndrome and OA, hyperlipidemia was not a risk factor for OA after adjusting for BMI and other factors [30]. We know that in the past, OA is generally considered to be a degenerative disease. With further research, it has been revealed that abnormal chondrocyte metabolism can change the inflammatory microenvironment and play a key role in the progression of cartilage degeneration in OA [31]. Studies had found that the synovial fluid of OA patients contained a high concentration of cholesterol [32]. When OA patients with high fat state, the expression of genes regulating cholesterol output was down-regulated, leading to continuous accumulation of cholesterol, resulting in continuous abnormal secretion of IL-1β and TNF-α [33]. Increased lipid levels also activated NLRP3 inflammasomes and lead to increased release of IL-1β [34]. Perhaps, inflammatory factors such as IL-1β and TNF-A explain the association. Il-1β is involved in various pathways leading to cartilage destruction, including inhibiting the production of collagen type II, stimulating the synthesis and breakdown of matrix metalloproteinases, and inhibiting the viability of chondrocytes [33, 35]. Adipose tissue can also affect the differentiation of bone marrow mesenchymal stem cells into osteoblasts or adipocytes through Wnt/β-catenin signaling pathway and PPARγ signaling pathway [36]. Therefore, a high level of blood lipid concentration will destroy the normal differentiation and metabolic activity of chondrocytes, thus promoting the occurrence of OA.

We also noted that serum uric acid levels were significantly higher in OA patients than in non-OA, but hyperuricemia was not confirmed for the occurrence and progression of OA disease. Our study result is similar to the previous clinical studies on blood lipids. The UA level of OA patients was higher in the female cohort [37]. Higher sUA levels were proved to associate with faster joint space narrowing and predicted the progression of joint structural changes [38]. Similarly, after adjusting for common factors such as age and BMI, serum UA level was no longer a risk factor for OA progression [39]. In vitro studies have confirmed that elevated synovial urate levels promoted OA, and the cartilage surface of OA injury is more conducive to urate deposition. Firstly, the deposition of monosodium urate (MSU) crystals on cartilage easily led to mechanical and inflammatory damage of chondrocytes [40]. Secondly, chondrocytes exposed to urate mainly activate NALP3 inflammasome, leading to the upregulation of inflammatory factors such as IL-1β [41, 42]. Denoble et al. also observed that urate concentration in synovial fluid with knee OA was correlated with the synovial IL-1β and IL-18 levels [42]. Therefore, the epidemiological relationship between hyperuricemia, hyperlipidemia, and OA in clinical studies is still controversial. Factors such as age, sex, and obesity appear to influence and alter the association between sUA, lipids, and OA outcomes. Our results also indicate that the levels of UA and lipid metabolic factors are significantly different between OA patients and non-OA patients, but the association needs to be further studied in the future.

Most arthritis symptoms in this cohort occurred before the diagnosis of pSS, with MCP, PIP, and wrist joints being the most commonly affected joints in pSS patients. When arthralgia was associated with pSS, the subjective symptoms of dry mouth and dry eyes were also more obvious. The ESSDAI score of patients with arthritis was significantly higher than that of patients without arthritis, suggesting not only that symptoms of arthritis are included in the assessment of disease activity status, but also that arthritis is associated with other systemic organ involvement, such as cutaneous vasculitis and muscle lesions. Previous studies on joint involvement in pSS patients had found the same results; pSS patients with AM had a higher risk of multi-system involvement, which was independently associated with renal involvement, RP, peripheral neuropathy, and cutaneous vasculitis [8]. Adrien Mirouse et al. also found that pSS patients with synovitis were more likely to have lymphadenopathy than patients without articular manifestations [10]. Compared with OA patients in this cohort, we saw that arthritis patients showed positive results of eye desiccation examination and salivary gland ECT, as well as higher positive rates of anti-SSA, anti-SSB, CCP antibody, and RF. The number of autoantibodies in pSS had already been shown to be positively correlated with the number of extraglandular manifestations [43]. Interestingly, we didn’t find any connection between arthritis and metabolic factors such as blood lipids, uric acid, or blood glucose. This may become the key point in the identification of arthralgia in pSS patients. When combined with arthritis, the disease symptoms and immunological characteristics of itself are more prominent, but when OA, the metabolic abnormalities are more obvious. Clinically, being able to identify the cause of arthralgia in patients with dryness has important implications for treatment options. Some studies had discovered patients treated with hydroxychloroquine (HCQ) had a lower incidence of arthritis [44]. Methotrexate (MTX) and rituximab (RTX) could effectively control the joint manifestations [10]. Cyclosporin A (CSA) treatment reduced the number of painful and swollen joints, which was an effective choice for pSS with joint involvement [45]. However, for osteoarthritis, the ACR guidelines did not recommend the use of HCQ, MTX, and other immunosuppressive agents [46], which had no significant effect on the improvement in joint pain and structural progress [47].

Therefore, it is necessary to identify the clinical phenotypes of arthralgia in pSS patients to make the appropriate treatment choices. To the best of our knowledge, this is the first time to compare the characteristics between arthritis and OA in pSS patients, to provide some differential evidence for the diagnosis of arthralgia in the future. Nevertheless, there are some limitations to our study. Firstly, it is a retrospective study that may have some limitations due to missing data. Secondly, it’*s* a small sample size. In the end, a prospective cohort study is needed to discuss the long-term outcomes of initial arthralgia in pSS patients and to explore the mechanisms by which arthralgia progresses to arthritis or OA.

## Supplementary Information

**Additional file 1. Table S1**. Characteristics of pSS patients with or without OA. **Table S2**. Characteristics of matched pSS patients with or without OA. **Table S3**. Characteristics of pSS patients with or without arthritis. **Table S4**. Characteristics of matched pSS patients with or without arthritis.

## Data Availability

The authors had full control of all primary data and agreed to allow the journal to review data if requested.

## References

[1] Mariette X, Criswell LA (2018). Primary Sjögren's syndrome. N Engl J Med.

[2] Parisis D, Chivasso C, Perret J, Soyfoo MS, Delporte C (2020). Current state of knowledge on primary Sjögren's syndrome, an autoimmune exocrinopathy. J Clin Med.

[3] Retamozo S, Acar-Denizli N, Rasmussen A, Horváth IF, Baldini C, Priori R (2019). Systemic manifestations of primary Sjögren'*s* syndrome out of the ESSDAI classification: prevalence and clinical relevance in a large international, multi-ethnic cohort of patients. Clin Exp Rheumatol.

[4] Chatzis LG, Stergiou IE, Goules AV, Pezoulas V, Tsourouflis G, Fotiadis D, Tzioufas AG, Voulgarelis M (2022). Clinical picture, outcome and predictive factors of lymphoma in primary Sjögren'*s* syndrome: results from a harmonized dataset (1981–2021). Rheumatology (Oxford).

[5] Haga HJ, Peen E (2007). A study of the arthritis pattern in primary Sjögren'*s* syndrome. Clin Exp Rheumatol.

[6] ter Borg EJ, Kelder JC (2016). Polyarthritis in primary Sjögren'*s* syndrome represents a distinct subset with less pronounced B cell proliferation a Dutch cohort with long-term follow-up. Clin Rheumatol.

[7] Castro-Poltronieri A, Alarcón-Segovia D (1983). Articular manifestations of primary Sjögren's syndrome. J Rheumatol.

[8] Vitali C, Del Papa N (2015). Pain in primary Sjögren'*s* syndrome. Best Pract Res Clin Rheumatol.

[9] Fauchais AL, Ouattara B, Gondran G, Lalloué F, Petit D, Ly K (2010). Articular manifestations in primary Sjögren'*s* syndrome: clinical significance and prognosis of 188 patients. Rheumatology (Oxford).

[10] Lazarus MN, Isenberg DA (2005). Development of additional autoimmune diseases in a population of patients with primary Sjögren'*s* syndrome. Ann Rheum Dis.

[11] Mirouse A, Seror R, Vicaut E, Mariette X, Dougados M, Fauchais AL (2019). Arthritis in primary Sjögren'*s* syndrome: characteristics, outcome and treatment from French multicenter retrospective study. Autoimmun Rev.

[12] Safiri S, Kolahi AA, Smith E, Hill C, Bettampadi D, Mansournia MA (2020). Global, regional and national burden of osteoarthritis 1990–2017: a systematic analysis of the Global Burden of Disease Study 2017. Ann Rheum Dis.

[13] Hunter DJ, March L, Chew M (2020). Osteoarthritis in 2020 and beyond: a lancet commission. Lancet.

[14] Vina ER, Kwoh CK (2018). Epidemiology of osteoarthritis: literature update. Curr Opin Rheumatol.

[15] Glyn-Jones S, Palmer AJ, Agricola R, Price AJ, Vincent TL, Weinans H, Carr AJ (2015). Osteoarthritis. Lancet.

[16] Allen KD, Thoma LM, Golightly YM (2022). Epidemiology of osteoarthritis. Osteoarthr Cartil.

[17] Lee YH, Tsou HK, Kao SL, Gau SY, Bai YC, Lin MC (2020). Patients with rheumatoid arthritis increased risk of developing osteoarthritis: a nationwide population-based cohort study in Taiwan. Front Med (Lausanne).

[18] Sakata K, Kaneko Y, Yasuoka H, Takeuchi T (2020). Association of radiographic findings in hand X-ray with clinical features and autoantibodies in patients with systemic sclerosis. Clin Rheumatol.

[19] Baron M, Lee P, Keystone EC (1982). The articular manifestations of progressive systemic sclerosis (scleroderma). Ann Rheum Dis.

[20] Aksoy A, Solmaz D, Can G, Cetin P, Balci A, Akar S (2016). Increased frequency of hand osteoarthritis in patients with primary Sjögren syndrome compared with systemic lupus erythematosus. J Rheumatol.

[21] Vitali C, Bombardieri S, Jonsson R, Moutsopoulos HM, Alexander EL, Carsons SE (2002). Classification criteria for Sjögren'*s* syndrome: a revised version of the European criteria proposed by the American-European Consensus Group. Ann Rheum Dis.

[22] Shiboski CH, Shiboski SC, Seror R, Criswell LA, Labetoulle M, Lietman TM (2017). 2016 American College of Rheumatology/European League Against Rheumatism Classification Criteria for Primary Sjögren'*s* Syndrome: a consensus and data-driven methodology involving three international patient cohorts. Arthritis Rheumatol.

[23] Joint Surgery Group (2018). Chinese Orthopaedics Association Osteoarthritis diagnosis and treatment guideline (2018). Chin J Orthop..

[24] Seror R, Ravaud P, Bowman SJ, Baron G, Tzioufas A, Theander E (2010). EULAR Sjogren’*s* syndrome disease activity index: development of a consensus systemic disease activity index for primary Sjogren’*s* syndrome. Ann Rheum Dis.

[25] Seror R, Ravaud P, Mariette X, Bootsma H, Theander E, Hansen A (2011). EULAR Sjogren’*s* Syndrome Patient Reported Index (ESSPRI): development of a consensus patient index for primary Sjogren’*s* syndrome. Ann Rheum Dis.

[26] Losina E, Weinstein AM, Reichmann WM, Burbine SA, Solomon DH, Daigle ME (2013). Lifetime risk and age at diagnosis of symptomatic knee osteoarthritis in the US. Arthritis Care Res (Hoboken).

[27] Coryell PR, Diekman BO, Loeser RF (2021). Mechanisms and therapeutic implications of cellular senescence in osteoarthritis. Nat Rev Rheumatol.

[28] O'Brien MS, McDougall JJ (2019). Age and frailty as risk factors for the development of osteoarthritis. Mech Ageing Dev.

[29] Zhou M, Guo Y, Wang D, Shi D, Li W, Liu Y, Yuan J, He M, Zhang X, Guo H, Wu T, Chen W (2017). The cross-sectional and longitudinal effect of hyperlipidemia on knee osteoarthritis: results from the Dongfeng-Tongji cohort in China. Sci Rep.

[30] Wang S, Pillinger MH, Krasnokutsky S, Barbour KE (2019). The association between asymptomatic hyperuricemia and knee osteoarthritis: data from the third National Health and Nutrition Examination Survey. Osteoarthr Cartil.

[31] Zheng L, Zhang Z, Sheng P, Mobasheri A (2021). The role of metabolism in chondrocyte dysfunction and the progression of osteoarthritis. Ageing Res Rev.

[32] Oliviero F, Lo Nigro A, Bernardi D, Giunco S, Baldo G, Scanu A, Sfriso P, Ramonda R, Plebani M, Punzi L (2012). A comparative study of serum and synovial fluid lipoprotein levels in patients with various arthritides. Clin Chim Acta.

[33] Farnaghi S, Crawford R, Xiao Y, Prasadam I (2017). Cholesterol metabolism in pathogenesis of osteoarthritis disease. Int J Rheum Dis.

[34] Duewell P, Kono H, Rayner KJ, Sirois CM, Vladimer G, Bauernfeind FG, Abela GS, Franchi L, Nuñez G, Schnurr M, Espevik T, Lien E, Fitzgerald KA, Rock KL, Moore KJ, Wright SD, Hornung V, Latz E (2010). NLRP3 inflammasomes are required for atherogenesis and activated by cholesterol crystals. Nature.

[35] Berenbaum F (2013). Osteoarthritis as an inflammatory disease (osteoarthritis is not osteoarthrosis!). Osteoarthr Cartil.

[36] Takada I, Kouzmenko AP, Kato S (2009). Molecular switching of osteoblastogenesis versus adipogenesis: implications for targeted therapies. Expert Opin Ther Targets.

[37] Kim SK, Kwak SG, Choe JY (2018). Serum uric acid level is not associated with osteoarthritis in Korean population: data from the Seventh Korea National Health and Nutrition Examination Survey 2016. Rheumatol Int.

[38] Krasnokutsky S, Oshinsky C, Attur M, Ma S, Zhou H, Zheng F, Chen M, Patel J, Samuels J, Pike VC, Regatte R, Bencardino J, Rybak L, Abramson S, Pillinger MH (2017). Serum urate levels predict joint space narrowing in non-gout patients with medial knee osteoarthritis. Arthritis Rheumatol.

[39] Go DJ, Kim DH, Kim JY, Guermazi A, Crema MD, Hunter DJ, Kim HA (2021). Serum uric acid and knee osteoarthritis in community residents without gout: a longitudinal study. Rheumatology (Oxford).

[40] Neogi T, Krasnokutsky S, Pillinger MH (2019). Urate and osteoarthritis: evidence for a reciprocal relationship. Joint Bone Spine.

[41] Martinon F, Pétrilli V, Mayor A, Tardivel A, Tschopp J (2006). Gout-associated uric acid crystals activate the NALP3 inflammasome. Nature.

[42] Denoble AE, Huffman KM, Stabler TV, Kelly SJ, Hershfield MS, McDaniel GE, Coleman RE, Kraus VB (2011). Uric acid is a danger signal of increasing risk for osteoarthritis through inflammasome activation. Proc Natl Acad Sci U S A.

[43] ter Borg EJ, Risselada AP, Kelder JC (2011). Relation of systemic autoantibodies to the number of extraglandular manifestations in primary Sjögren's syndrome: a retrospective analysis of 65 patients in the Netherlands. Semin Arthritis Rheum.

[44] Demarchi J, Papasidero S, Medina MA, Klajn D, Chaparro Del Moral R, Rillo O, Martiré V, Crespo G, Secco A, Catalan Pellet A, Amitrano C, Crow C, Asnal C, Pucci P, Caeiro F, Benzanquen N, Pirola JP, Mayer M, Zazzetti F, Velez S, Barreira J, Tamborenea N, Santiago L, Raiti L (2017). Primary Sjögren'*s* syndrome: extraglandular manifestations and hydroxychloroquine therapy. Clin Rheumatol.

[45] Kedor C, Zernicke J, Hagemann A, Gamboa LM, Callhoff J, Burmester GR, Feist E (2016). A phase II investigator-initiated pilot study with low-dose cyclosporine A for the treatment of articular involvement in primary Sjögren'*s* syndrome. Clin Rheumatol.

[46] Kolasinski SL, Neogi T, Hochberg MC, Oatis C, Guyatt G, Block J, Callahan L, Copenhaver C, Dodge C, Felson D, Gellar K, Harvey WF, Hawker G, Herzig E, Kwoh CK, Nelson AE, Samuels J, Scanzello C, White D, Wise B, Altman RD, DiRenzo D, Fontanarosa J, Giradi G, Ishimori M, Misra D, Shah AA, Shmagel AK, Thoma LM, Turgunbaev M, Turner AS, Reston J (2020). 2019 American College of Rheumatology/Arthritis Foundation Guideline for the Management of Osteoarthritis of the Hand, Hip, and Knee. Arthritis Care Res (Hoboken).

[47] Ferrero S, Wittoek R, Allado E, Cruzel C, Fontas E, Breuil V, Ziegler L, Kremer J, Loeuille D, Roux CH (2021). Methotrexate treatment in hand osteoarthritis refractory to usual treatments: a randomised, double-blind, placebo-controlled trial. Semin Arthritis Rheum.

