# Supplementary material for: Osteoarthritis or arthritis? Toward understanding of primary Sjögren’s syndrome patients with arthralgia
Source: J Orthop Surg Res. 2023 Jan 16;18:41. doi: 10.1186/s13018-023-03513-1 (PMC9841668; doi:10.1186/s13018-023-03513-1)
Supplement: Supplementary file 1 — Additional file 1. Table S1. Characteristics of pSS patients with or without OA. Table S2. Characteristics of matched pSS patients with or without OA. Table S3. Characteristics of pSS patients with or without arthritis. Table S4. Characteristics of matched pSS patients with or without arthritis. [file 13018_2023_3513_MOESM1_ESM.docx]

**Osteoarthritis or arthritis? Towards understanding of primary Sjögren’s Syndrome patients with arthralgia**

**Authors**: Ronglin Gao^1^, Jincheng Pu^1^, Zhenzhen Wu^1^, Jianping Tang^1^, Xuan Wang*^1^

**Affiliation:**

1 Department of Rheumatology and Immunology, Tongji Hospital, School of Medicine, Tongji University, No. 389 Xincun Road, Shanghai 200065, China.

***Corresponding authors:** Xuan Wang.

Xuan Wang: Department of Rheumatology and Immunology, Tongji Hospital, School of Medicine, Tongji University, No. 389 Xincun Road, Shanghai 200065, China. E-mail: [xuan2018@tongji.edu.cn](mailto:xuan2018@tongji.edu.cn). Tel: +8613918745569

**Supplementary material 1**

**Table S1** Characteristics of pSS patients with or without OA

| **Variable** | pSS-OA | pSS-nOA | p Value^1^ | p Value^2^ |
| --- | --- | --- | --- | --- |
|  | n=186 | n=182 |  |  |
| **Female** (n, %) | 172(92.5) | 166(91.2) | 0.659 | 0.751 |
| **Age** (≥65 year) (n, %) | 71(38.2) | 41(22.5) | 0.001 | 0.024 |
| **Disease duration** (year) M (IQR) | 5(2-10) | 3(1-10) | 0.034 | 0.132 |
| **Clinical manifestation** (n, %) |  |  |  |  |
| Dry mouth | 164(88.2) | 150(82.4) | 0.119 | 0.247 |
| Dry eye | 134(72.8) | 121(66.9) | 0.215 | 0.376 |
| Mouth ulcers | 52(28.0) | 37(20.3) | 0.088 | 0.205 |
| Dental caries and dentures | 114(61.3) | 102(56.0) | 0.308 | 0.419 |
| Swollen parotid gland | 20(10.8) | 20(11.0) | 0.942 | 0.982 |
| Rash | 34(18.3) | 45(24.7) | 0.133 | 0.251 |
| Renault phenomenon | 22(11.8) | 32(17.6) | 0.119 | 0.247 |
| Joint pain | 108(58.1) | 54(29.7) | <0.001 | 0.024 |
| Fatigue | 35(18.8) | 43(23.6) | 0.260 | 0.402 |
| **ESSDAI score** M (IQR) | 4(2-8) | 5(3-10) | 0.006 | 0.074 |
| **ESSDAI domains** (n, %) |  |  |  |  |
| Constitutional | 1(0.5) | 1(0.5) | 1.000 | 1.000 |
| Lymphadenopathy | 8(4.3) | 11(6.0) | 0.451 | 0.579 |
| Glandular | 22(11.8) | 17(9.4) | 0.461 | 0.579 |
| Articular | 24(12.9) | 17(9.4) | 0.279 | 0.402 |
| Cutaneous | 5(2.7) | 13(7.1) | 0.048 | 0.160 |
| Pulmonary | 35(18.8) | 42(23.5) | 0.278 | 0.402 |
| Renal | 7(3.8) | 6(3.4) | 0.833 | 0.907 |
| Muscular | 4(2.2) | 3(1.6) | 1.000 | 1.000 |
| Peripheral nervous system | 6(3.2) | 11(6.0) | 0.199 | 0.361 |
| Central nervous system | 0(0.0) | 1(0.5) | 0.495 | 0.594 |
| Hematological | 109(58.6) | 123(67.6) | 0.075 | 0.191 |
| Biological | 103(55.7) | 114(64.8) | 0.078 | 0.191 |
| **Lab data** |  |  |  |  |
| Hemoglobin (g/L), x±s | 119±14.8 | 115±18.8 | 0.030 | 0.132 |
| A/G | 1.3±0.3 | 1.2±0.3 | 0.012 | 0.098 |
| Globulin (g/L), x±s | 31.8±7.5 | 34.5±11.0 | 0.008 | 0.078 |
| Albumin (g/L), x±s | 38.2±6.0 | 37.1±5.1 | 0.058 | 0.167 |
| K^+^ (mmol/L), x±s | 3.8±0.3 | 3.7±0.4 | 0.015 | 0.105 |
| Uric acid (umol/L), M (IQR) | 276(234.7-328.3) | 261(218.0-317.0) | 0.030 | 0.132 |
| TC (mmol/L), M (IQR) | 4.6(4.0-5.3) | 4.5(3.7-4.9) | 0.025 | 0.132 |
| TG (mmol/L), M (IQR) | 1.3(0.9-1.7) | 1.3(0.9-1.4) | 0.287 | 0.402 |
| HDL (mmol/L), M (IQR) | 1.2(1.0-1.4) | 1.2(0.9-1.3) | 0.064 | 0.174 |
| FBG (mmol/L), x±s | 5.0±1.0 | 4.9±1.0 | 0.537 | 0.627 |
| C3 (g/L), x±s | 1.0±0.2 | 0.9±0.2 | 0.256 | 0.402 |
| C4 (g/L), x±s | 0.2±0.1 | 0.19±0.1 | 0.004 | 0.065 |
| ANA positive (n, %) | 113(61.1) | 131(72.0) | 0.027 | 0.132 |
| Anti-SSA positive (n, %) | 107(57.8) | 123(67.6) | 0.054 | 0.165 |
| Anti-SSB positive (n, %) | 48(25.9) | 53(29.1) | 0.497 | 0.594 |
| Anti-CCP positive (n, %) | 5(3.1) | 4(2.8) | 0.868 | 0.925 |
| Increased RF (n, %) | 38(22.9) | 45(28.3) | 0.265 | 0.402 |
| IgA (g/L), x±s | 2.9±1.4 | 3.3±2.9 | 0.049 | 0.160 |
| IgM (g/L), x±s | 1.4±1.4 | 1.6±2.2 | 0.444 | 0.579 |
| IgG (g/L), x±s | 15.5±6.0 | 16.7±7.2 | 0.035 | 0.132 |
| IgG4 (g/L), x±s | 0.6±1.8 | 0.5±0.8 | 0.696 | 0.775 |
| Increased CRP (n, %) | 15(8.3) | 23(13.3) | 0.133 | 0.251 |
| Increased ESR (n, %) | 51(28.3) | 60(36.1) | 0.121 | 0.247 |
| Decreased 25-(OH)D (n, %) | 28(17.5) | 18(12.5) | 0.226 | 0.382 |

p Value^1^ means the unadjusted P value; p Value^2^ means the adjusted P value using the Benjamini-Hochberg method.

Abbreviation: OA: osteoarthritis; A/G: albumin/globulin; UA: uric acid; TC: total cholesterol; TG: triglycerides; HDL: high density lipoprotein; FBG: fasting blood glucose; C3: complement 3; C4: complement 4; ANA: antinuclear antibodies; CCP: cyclic citrullinated peptide; RF: rheumatoid factor; CRP: C-reactive protein; ESR: erythrocyte sedimentation rate; 25-(OH) D: 25-hydroxy vitamin D.

**Supplementary material 2**

**Table S2** Characteristics of matched pSS patients with or without OA

| **Variable** | pSS without OA | pSS with OA | p Value |
| --- | --- | --- | --- |
|  | n=96 | n=96 |  |
| **Female** (n, %) | 90(93.8) | 92(95.8) | 0.745 |
| **Age,** year, x±s | 59.5±8.63 | 59.7±8.28 | 0.898 |
| **Disease duration** (year) M (IQR) | 5(2-10) | 5(2-10) | 1.000 |
| **ESSDAI score** M (IQR) | 4.5(2-10) | 4.0(2-10) | 0.546 |
| **Clinical manifestation** (n, %) |  |  |  |
| Dry mouth | 85(88.5) | 84(87.5) | 1.000 |
| Dry eye | 68(70.8) | 71(74.0) | 0.743 |
| Swollen parotid gland | 10(10.4) | 10(10.4) | 1.000 |
| Renault phenomenon | 15(15.6) | 11(11.5) | 0.527 |
| Joint pain | 24(25.0) | 54(56.3) | <0.001 |
| Articular | 8(8.3) | 13(13.5) | 0.355 |
| Lymphadenopathy | 3(3.1) | 7(7.3) | 0.330 |
| Cutaneous | 7(7.3) | 4(4.2) | 0.535 |
| Pulmonary | 18(18.8) | 26(27.1) | 0.229 |
| Renal | 2(2.1) | 1(1.0) | 1.000 |
| Muscular | 3(3.1) | 0(0.0) | 0.244 |
| Peripheral nervous system | 9(9.4) | 1(1.0) | 0.023 |
| Central nervous system | 0(0.0) | 1(0.3) | 1.000 |
| Hematological | 58(60.4) | 54(56.3) | 0.661 |
| Biological | 59(61.5) | 56(58.3) | 0.768 |
| **Lab data** |  |  |  |
| Hemoglobin (g/L), x±s | 118±17.9 | 121±11.7 | 0.306 |
| A/G | 1.22±0.3 | 1.27±0.3 | 0.233 |
| Globulin (g/L), x±s | 33.5±10.7 | 31.5±6.74 | 0.133 |
| Uric acid (umol/L), M (IQR) | 261(216-317) | 267(231-327) | 0.943 |
| TC (mmol/L), M (IQR) | 4.51(3.9-5.3) | 4.58(4.1-5.3) | 0.391 |
| TG (mmol/L), M (IQR) | 1.26(0.9-1.4) | 1.32(0.9-1.7) | 0.452 |
| HDL (mmol/L), M (IQR) | 1.18(1.0-1.3) | 1.19(1.0-1.4) | 0.452 |
| FBG (mmol/L), x±s | 4.97±0.8 | 4.89±0.8 | 0.442 |
| C3 (g/L), x±s | 0.9±0.2 | 1.0±0.2 | 0.298 |
| C4 (g/L), x±s | 0.19±0.1 | 0.2±0.1 | 0.152 |
| ANA positive (n, %) | 66(68.8) | 62(64.6) | 0.646 |
| IgA (g/L), x±s | 3.3±2.2 | 2.8±1.3 | 0.067 |
| IgM (g/L), x±s | 1.4±1.0 | 1.4±1.3 | 0.751 |
| IgG (g/L), x±s | 16.1±6.1 | 15.1±5.4 | 0.256 |

Abbreviation: OA: osteoarthritis; A/G: albumin/globulin; UA: uric acid; TC: total cholesterol; TG: triglycerides; HDL: high density lipoprotein; FBG: fasting blood glucose; C3: complement 3; C4: complement 4; ANA: antinuclear antibodies; IgA: Immunoglobulin A.

**Supplementary material 3**

**Table S3** Characteristics of pSS patients with or without arthritis

| **Variable** | pSS with arthritis | pSS without arthritis | p Value^1^ | p Value^2^ |
| --- | --- | --- | --- | --- |
|  | n=41 | n=327 |  |  |
| **Female** (n, %) | 38(92.7) | 300(91.7) | 1.000 | 1.000 |
| **Age** (≥65 year) (n, %) | 13(31.7) | 99(30.3) | 0.851 | 1.000 |
| **Disease duration** (year) M (IQR) | 4(1-8) | 4(2-10) | 0.173 | 0.982 |
| **ESSDAI score** M (IQR) | 7(4-9.5) | 4(2-10) | 0.011 | 0.231 |
| **Clinical manifestation** (n, %) |  |  |  |  |
| Dry mouth | 38(92.7) | 276(84.4) | 0.159 | 0.982 |
| objective | 21(91.3) | 201(91.8) | 1.000 | 1.000 |
| Dry eye | 28(68.3) | 227(70.1) | 0.817 | 1.000 |
| objective | 12(85.7) | 70(82.4) | 1.000 | 1.000 |
| Swollen parotid gland | 3(7.3) | 37(11.3) | 0.611 | 1.000 |
| Renault phenomenon | 4(9.8) | 50(15.3) | 0.346 | 1.000 |
| Joint pain | 36(87.8) | 126(38.5) | <0.001 | 0.042 |
| Lymphadenopathy | 1(2.4) | 18(5.5) | 0.644 | 1.000 |
| Cutaneous | 3(7.3) | 15(4.6) | 0.704 | 1.000 |
| Pulmonary | 9(22.0) | 68(21.0) | 0.887 | 1.000 |
| Renal | 2(4.9) | 11(3.4) | 0.972 | 1.000 |
| Muscular | 3(7.3) | 4(1.2) | 0.037 | 0.518 |
| Peripheral nervous system | 2(4.9) | 15(4.6) | 1.000 | 1.000 |
| Central nervous system | 0(0.0) | 1(0.3) | 1.000 | 1.000 |
| Hematological system | 25(61.0) | 207(63.3) | 0.772 | 1.000 |
| **Lab data** |  |  |  |  |
| Hemoglobin (g/L), x±s | 118±17.3 | 117±17.0 | 0.653 | 1.000 |
| A/G | 1.2±0.3 | 1.2±0.3 | 0.233 | 1.000 |
| Globulin (g/L), x±s | 33.1±6.3 | 33.2±9.8 | 0.980 | 1.000 |
| K^+^ (mmol/L), x±s | 3.8±0.3 | 3.8±0.4 | 0.059 | 0.619 |
| Uric acid (umol/L), M (IQR) | 273(209-331) | 267(230-316) | 0.881 | 1.000 |
| TC (mmol/L), M (IQR) | 4.4(3.9-5.3) | 4.5(3.8-5.3) | 0.951 | 1.000 |
| TG (mmol/L), M (IQR) | 1.1(0.8-1.7) | 1.2(0.9-1.7) | 0.246 | 1.000 |
| HDL (mmol/L), M (IQR) | 1.1(0.9-1.6) | 1.2(0.9-1.4) | 0.817 | 1.000 |
| FBG (mmol/L), x±s | 4.8±0.8 | 5.0±1.0 | 0.297 | 1.000 |
| C3 (g/L), x±s | 1.0±0.2 | 0.9±0.2 | 0.187 | 0.982 |
| C4 (g/L), x±s | 0.2±0.1 | 0.2±0.1 | 0.435 | 1.000 |
| ANA positive (n, %) | 28(68.3) | 216(66.3) | 0.795 | 1.000 |
| Anti-SSA (n, %) | 24(58.5) | 206(63.2) | 0.563 | 1.000 |
| Anti-SSB (n, %) | 9(22.0) | 92(28.2) | 0.398 | 1.000 |
| Anti-CCP (n, %) | 1(2.9) | 8(3.0) | 1.000 | 1.000 |
| Increased RF (n, %) | 14(36.8) | 69(24.0) | 0.090 | 0.756 |
| IgA (g/L), x±s | 2.9±1.1 | 3.1±2.4 | 0.540 | 1.000 |
| IgM (g/L), x±s | 1.3±0.9 | 1.5±2.0 | 0.511 | 1.000 |
| IgG (g/L), x±s | 16.8±5.4 | 16.1±6.8 | 0.573 | 1.000 |
| IgG4 (g/L), x±s | 0.5±0.6 | 0.6±1.5 | 0.956 | 1.000 |
| Increased CRP (n, %) | 3(7.7) | 35(11.1) | 0.702 | 1.000 |
| Increased ESR (n, %) | 12(29.3) | 99(32.5) | 0.682 | 1.000 |
| Decreased 25-(OH)D (n, %) | 6(18.8) | 40(14.7) | 0.732 | 1.000 |

p Value^1^ means the unadjusted P value; p Value^2^ means the adjusted P value using the Benjamini-Hochberg method.

Abbreviation: A/G: albumin/globulin; UA: uric acid; TC: total cholesterol; TG: triglycerides; HDL: high density lipoprotein; FBG: fasting blood glucose; C3: complement 3; C4: complement 4; ANA: antinuclear antibodies; CCP: cyclic citrullinated peptide; RF: rheumatoid factor; CRP: C-reactive protein; ESR: erythrocyte sedimentation rate; 25-(OH) D: 25-hydroxy vitamin D.

**Supplementary material 4**

**Table S4** Characteristics of matched pSS patients with or without arthritis

| **Variable** | pSS without arthritis | pSS with arthritis | p Value |
| --- | --- | --- | --- |
|  | n=98 | n=32 |  |
| **Female** (n, %) | 94(95.9) | 29(90.6) | 0.483 |
| **Age,** year, x±s | 57.6±9.67 | 58.7±11.80 | 0.645 |
| **Disease duration** (year) M (IQR) | 4(2-7) | 4.5(2-10) | 0.800 |
| **ESSDAI score** M (IQR) | 3.5(2-8) | 6.5(3-8) | 0.157 |
| **Clinical manifestation** (n, %) |  |  |  |
| Dry mouth | 83(84.7) | 30(93.8) | 0.309 |
| Dry eye | 70(71.4) | 24(75.0) | 0.933 |
| Swollen parotid gland | 10(10.2) | 2(6.3) | 0.750 |
| Renault phenomenon | 13(13.3) | 4(12.5) | 1.000 |
| Joint pain | 37(37.8) | 29(90.6) | <0.001 |
| Lymphadenopathy | 5(5.1) | 0(0.0) | 0.439 |
| Cutaneous | 4(4.1) | 2(6.3) | 0.982 |
| Pulmonary | 17(17.3) | 7(21.9) | 0.756 |
| Renal | 1(1.0) | 1(3.1) | 0.990 |
| Muscular | 1(1.0) | 2(6.3) | 0.302 |
| Peripheral nervous system | 3(3.1) | 2(6.3) | 0.776 |
| Central nervous system | 0(0.0) | 0(0.0) | 1.000 |
| Hematological | 59(60.2) | 18(56.3) | 0.851 |
| Biological | 57(58.2) | 17(53.1) | 0.769 |
| **Lab data** |  |  |  |
| Hemoglobin (g/L), x±s | 119±14.7 | 121±16.5 | 0.640 |
| A/G | 1.28±0.3 | 1.17±0.2 | 0.045 |
| Globulin (g/L), x±s | 33.1±10.8 | 33.4±5.71 | 0.866 |
| Uric acid (umol/L), M (IQR) | 258(216-316) | 276(220-342) | 0.394 |
| TC (mmol/L), M (IQR) | 4.51(4.0-5.2) | 4.61(4.1-5.5) | 0.743 |
| TG (mmol/L), M (IQR) | 1.33(0.9-1.7) | 1.23(0.9-1.7) | 0.053 |
| HDL (mmol/L), M (IQR) | 1.18(1.0-1.4) | 1.11(1.0-1.4) | 0.752 |
| FBG (mmol/L), x±s | 4.87±0.6 | 4.63±0.3 | 0.008 |
| C3 (g/L), x±s | 0.94±0.2 | 1.0±0.2 | 0.127 |
| C4 (g/L), x±s | 0.19±0.1 | 0.21±0.1 | 0.194 |
| ANA positive (n, %) | 61(62.6) | 22(68.8) | 0.650 |
| IgA (g/L), x±s | 2.81±1.2 | 3.02±1.0 | 0.328 |
| IgM (g/L), x±s | 1.56±1.3 | 1.30±0.8 | 0.181 |
| IgG (g/L), x±s | 15.4±5.5 | 16.7±5.0 | 0.244 |

Abbreviation: A/G: albumin/globulin; UA: uric acid; TC: total cholesterol; TG: triglycerides; HDL: high density lipoprotein; FBG: fasting blood glucose; C3: complement 3; C4: complement 4; ANA: antinuclear antibodies; IgA: Immunoglobulin A.
